# Pathogen prevalence, feature composition and cross-centre generalisability of machine learning diagnostic models for multi-pathogen respiratory infection

**DOI:** 10.3389/fpubh.2026.1961816

**Published:** 2026-09-16

**Authors:** Fengmiao Hu, Xingyu Zhou, Lijun Zhou, Zhirui Li, Shuang Dong, Chongkun Xiao

**Affiliations:** Sichuan Center for Disease Control and Prevention, Chengdu, China

**Keywords:** cross-centre validation, feature composition, machine learning, multi-label classification, multi-pathogen diagnosis, respiratory infection

## Abstract

**Objective:**

To evaluate the predictive information contained in the restricted surveillance feature set (demographic, temporal and specimen variables) for respiratory pathogen identification, and to examine factors associated with model performance.

**Methods:**

We retrospectively analysed 24,689 acute respiratory infection cases from seven sentinel hospitals in Sichuan, China; after quality control, 21,395 samples with complete 21-pathogen testing were included. Per-pathogen multi-label binary classifiers were trained using 15 features available at presentation; logistic regression, random forest, back-propagation neural network and XGBoost were compared under an identical split and threshold-selection protocol, with thresholds derived from out-of-fold probabilities. Label-definition sensitivity, leave-one-hospital-out cross-validation and prevalence-performance association were analysed.

**Results:**

Random forest and XGBoost performed comparably (XGBoost the primary model), with Macro-F1 0.1553 (95% CI 0.104–0.2091), 0.2202 for common pathogens and Macro-AUC 0.7702; the four-algorithm spread was 0.0611. Log-prevalence correlated strongly with per-pathogen F1 (*r* = 0.851); the seven rare pathogens reached Macro-F1 0.0255. Leave-one-hospital-out validation gave Macro-F1 0.1073 ± 0.0289 (−30.9%) and Macro-AUC 0.6841 (−11.2%). Feature ablation showed temporal and non-temporal features were largely redundant (Macro-F1 0.1153 vs. 0.1147; AUC 0.6892/0.6865 vs. 0.7702). Treating untested as negative lowered Macro-AUC to 0.7653, whereas simulated false-negative label contamination had minimal effect.

**Conclusion:**

Models built on the restricted surveillance feature set showed limited diagnostic performance and were not demonstrated suitable for clinical deployment. Performance was strongly associated with pathogen prevalence and constrained by limited feature informativeness, while algorithm choice contributed little and cross-centre generalisability was limited. Priorities include multimodal feature integration, rare-pathogen accrual and prospective multicentre external validation.

## 1 Introduction

Respiratory infections consistently rank among the most prevalent infectious diseases in China, and machine learning models based on hospital clinical data centres have already been used to detect and predict suspected respiratory infectious diseases (1). The widespread adoption of multiplex nucleic acid testing has expanded the spectrum of identifiable pathogens, and co-infections are now recognised more frequently (2). Identifying the likely causative pathogen before laboratory results become available is therefore of practical value for guiding empirical treatment and the rational use of antimicrobials.

Machine learning offers a potential technical route for this task. Ge et al. developed an auxiliary diagnostic model for paediatric influenza A and B based on haematological data using logistic regression and gradient-boosted decision trees, achieving an AUC of 0.902 for influenza B (3); Mao et al. reported an AUC of 0.796 for a gradient-boosting model of adenovirus pneumonia in children (4); and Su et al. developed and validated a machine-learning diagnostic system covering 22 pathogens using 134,500 samples (5). Other machine-learning approaches have been applied to respiratory virus infection prediction (6) and to refractory *Mycoplasma pneumoniae* pneumonia diagnosis (7). Nevertheless, previous studies share three limitations that may lead to overestimation of model performance: most focused on binary classification of a single pathogen and neglected the multi-label nature of co-infection (8); the inputs relied on in-hospital information such as serological markers (9) and imaging findings (10) that are often unavailable at the first point of care; and validation was predominantly based on single-centre random splits without external cross-centre validation (11). In addition, the cost of treating “untested” items as negative has not been quantitatively evaluated.

The objective of this study was therefore to quantify the pathogen-discriminative information contained in a restricted surveillance feature set (demographic, temporal and specimen variables available at the time of consultation) for respiratory pathogen identification, and to examine the factors associated with model performance under routine surveillance data conditions. Quantifying how much information routinely collected surveillance variables carry is a prerequisite for deciding where richer predictors are needed before such models could support empirical treatment decisions.

## 2 Materials and methods

This was a diagnostic prediction model development study (12) based on retrospective surveillance data, with development and validation performed using random splits within the same dataset and leave-one-hospital-out cross-validation as quasi-external validation. The purpose of this study was not to deliver a diagnostic tool ready for clinical deployment, but to quantify how much pathogen-discriminative information is contained in a surveillance feature set of only routinely recorded demographic, temporal and specimen variables, and to examine which factors are associated with model performance; therefore, no clinical deployment threshold was predefined.

Specifically, the study analysed 21,395 samples with complete testing for all 21 pathogens from seven sentinel hospitals in Sichuan Province. Four representative algorithms were compared within a multi-label framework under an identical split and threshold-selection protocol; label-definition sensitivity analysis, a cohort-fixed contamination simulation, feature-group ablation, permutation-importance analysis, calibration assessment, leave-one-hospital-out cross-validation and a prevalence-performance association analysis were used to characterise the sources of model performance. Because symptoms, clinical signs, vital parameters, comorbidities, vaccination status and epidemiological exposures were not recorded in the surveillance database, the present study should be read as an evaluation of the predictive information contained in this restricted feature set rather than a general assessment of achievable pathogen-level diagnostic prediction.

### 2.1 Study population and sample inclusion

The study included 24,689 acute respiratory infection cases reported between January 2024 and July 2026 by seven national sentinel hospitals for respiratory pathogen surveillance in Sichuan Province. After demographic consistency quality control (excluding 1,133 records with logical contradictions between age and occupation) and the exclusion of 2,161 samples with any untested pathogen, 21,395 cases entered the primary analysis (52.7% male; mean age 26.6 ± 27.6 years). Because “untested” is neither positive nor negative, complete-case analysis was used to ensure label authenticity.

We deliberately did not use multiple imputation or weak-label learning for the 2,161 excluded records, for three reasons. First, missingness was not random: ‘untested’ status clustered in younger and milder patients and was strongly associated with disease severity (Section 3.1), violating the missing-at-random assumption required by multiple imputation (13). Second, ‘untested’ is a documentation artefact of the surveillance workflow rather than a noisy or weak label: weakly supervised learning assumes that cheap, imperfect labels can be exploited at scale (14), whereas here the complete-case labels are authentic PCR results and the excluded records simply lack them; coding ‘untested’ as negative degraded ranking performance (Definition A, Section 3.4). Third, complete-case analysis preserved the authenticity of all 21 labels in the analytic cohort. We nonetheless acknowledge that the exclusion may affect model calibration: because the analytic cohort was enriched for severe cases, predicted probabilities fitted on these samples may not be well calibrated for predominantly mild outpatient populations (Section 3.8 and Section 5).

This study was approved by the Ethics Review Committee of Sichuan Provincial Center for Disease Control and Prevention (SCCDCIRB 2026–026). As a retrospective analysis, informed consent was waived; the ethics approval was obtained retrospectively, after data collection had commenced, and covered the analysis of de-identified retrospective surveillance records (Table 1).

**Table 1:** Flow diagram of sample inclusion and exclusion: from 24,689 reported acute respiratory infection records to 21,395 analysed cases after demographic quality control and exclusion of partially tested samples.

Step	Inclusion/exclusion criteria	No. of samples
Raw surveillance data	ARI cases reported by 7 sentinel hospitals	24,689
Demographic consistency QC	Excluding records with age–occupation contradictions	–1,133
After QC	Passing demographic consistency check	23,556
Excluding partial-testing samples	Any pathogen untested	–2,161
Primary analysis samples	Complete 21-pathogen testing with intact labels	21,395

Samples with incomplete pathogen testing were excluded from the primary analysis to avoid treating “untested” as negative and introducing false-negative labels; the impact of this exclusion is examined in Section 3.4.

### 2.2 Pathogen detection

All samples were tested by the Sichuan Provincial Center for Disease Control and Prevention and seven prefecture-level CDCs (cities and autonomous prefectures). Within 3 working days of specimen receipt, each laboratory performed multiplex real-time polymerase chain reaction (PCR) testing (representative instruments: Bio-Rad CFX96 / Thermo Fisher ABI QuantStudio 7 real-time PCR systems). Nucleic acid was extracted using fully automated magnetic-bead extraction instruments (representative reagents: Tianlong nucleic acid extraction kit and Tianlong GeneRotex96 fully automated nucleic acid extractor). Commercial multiplex respiratory pathogen kits were used for detection. Representative reagents included the 23-pathogen respiratory pre-mixed nucleic acid detection kit (Jiangsu Bioperfectus Technologies Co., Ltd.)^1^ and the 27-pathogen respiratory pathogen real-time fluorescence quantitative PCR detection kit (Beijing Zhuocheng Huisheng Biotechnology Co., Ltd.;^2^ product A786AA),^3^ covering both routine and recommended testing categories. Per-pathogen positive rates are shown in Table 2. From the pathogens detectable with these commercial kits, 21 species with sufficient detection coverage and adequate positive sample counts were selected for inclusion in the present diagnostic modelling analysis.

**Table 2:** Detection panel and positive rates of the 21 respiratory pathogens (*n* = 21,395), by category (10 viruses, 6 bacteria, 2 fungi, 3 Chlamydia/Mycoplasma).

Category	Pathogen	Abbreviation	Positive cases	Positive rate
Viruses	SARS-CoV-2	SARS-CoV-2	835	3.90%
Viruses	Influenza virus	IV	2,126	9.94%
Viruses	Respiratory syncytial virus	RSV	864	4.04%
Viruses	Adenovirus	AdV	672	3.14%
Viruses	Human metapneumovirus	hMPV	437	2.04%
Viruses	Parainfluenza virus	PIV	691	3.23%
Viruses	Common coronavirus	CoV	574	2.68%
Viruses	Bocavirus	BoV	176	0.82%
Viruses	Rhinovirus	RV	1,911	8.93%
Viruses	Enterovirus	EV	770	3.60%
Bacteria	*Streptococcus pneumoniae*	SP	2,314	10.82%
Bacteria	*Haemophilus influenzae*	HI	3,296	15.41%
Bacteria	Group A Streptococcus	GAS	218	1.02%
Bacteria	*Bordetella pertussis*	BP	57	0.27%
Bacteria	*Legionella* spp.	LP	7	0.03%
Bacteria	*Klebsiella pneumoniae*	KP	617	2.88%
Fungi	*Aspergillus*	AF	80	0.37%
Fungi	*Cryptococcus*	CN	6	0.03%
Chlamydia/Mycoplasma	*Mycoplasma pneumoniae*	MP	261	1.22%
Chlamydia/Mycoplasma	*Chlamydia psittaci*	CP	10	0.05%
Chlamydia/Mycoplasma	*Chlamydia pneumoniae*	Cpn	73	0.34%

### 2.3 Multi-label task formulation

Co-infection accounted for 17.8% of the cohort, and pathogen labels were therefore not mutually exclusive. The task was formulated as multi-label classification, in which an independent binary classifier was trained for each of the 21 pathogens using a one-vs-rest strategy, allowing the performance of each pathogen to be evaluated and attributed separately.

### 2.4 Feature engineering

Features were selected according to the principle of using only information available at the time of consultation that does not depend on any laboratory result. A total of 15 features were constructed: age, log-transformed age, sex, severity, month, quarter, weekday, day of year, sine of month, cosine of month, winter indicator, illness duration, occupation, specimen type and specimen source. The surveillance database does not record symptoms, clinical signs, vital parameters, comorbidities, vaccination status or epidemiological exposures; these variables were therefore unavailable rather than deliberately excluded. Consequently, seven of the 15 features are overlapping representations of calendar time and seasonality (month, quarter, weekday, day of year, sine/cosine of month and a winter indicator), and the model is expected to capture seasonal epidemiological priors more readily than pathogen-specific patient characteristics. Month was encoded as periodic sine/cosine features, and the three categorical variables were ordinal-encoded. Missing values were handled as follows: age was imputed with the training-set median, illness duration with 0, categorical variables were treated as a separate category, and the severity indicator was treated as absent. Because several temporal features are mechanically inter-correlated (month, quarter, day of year, winter, sine and cosine of month), we computed variance inflation factors (VIF): month (VIF = 164.3), day of year (153.1) and quarter (20.1) exceeded the conventional threshold of 10, confirming multicollinearity among the calendar encodings, while the remaining features had VIF < 4, except age and log-age (10.1 − 10.4), which are mathematically related by construction (15). VIF is a linear-model diagnostic and does not directly apply to tree ensembles, which are invariant to monotone transformations and robust to redundant predictors; we therefore evaluated whether this redundancy distorted learning using permutation importance (Section 3.6) and feature-group ablation (Section 3.7). Because the algorithm comparison also includes L2-regularised logistic regression, whose linear coefficients are directly affected by multicollinearity, we performed an additional robustness check: the logistic regression was re-run on a reduced feature set with the four features exceeding VIF = 10 removed (month, day of year, quarter and age; the reduced set retained log-age). The reduced-set maximum VIF was 3.06 (mean 1.6), and the reduced-set logistic regression achieved a Macro-F1 of 0.1318 and a Macro-AUC of 0.7292, compared with 0.1358 and 0.7368 for the full 15-feature set (Section 3.2). The stability of the logistic-regression performance after removing the collinear encodings indicates that the multicollinearity among calendar variables did not distort the linear model’s predictions either, consistent with the L2 regularisation applied. Beyond predictive performance, we also inspected the logistic-regression coefficients themselves. After removing the four high-VIF calendar features, the signs of the coefficients of the retained core predictors (e.g., age, severity and specimen source) remained stable, with no sign flips or order-of-magnitude changes, although the magnitudes of some coefficients changed modestly; the direction of the meaningful predictors was preserved. Because the focus of this study is multi-label predictive discrimination rather than effect estimation, the full coefficient table is not presented in the main text; the coefficient estimates are available from the analysis code and Supplementary materials.

The overall workflow is summarised in Figure 1. From 24,689 reported cases, 21,395 complete-case samples (Definition B) were analysed and randomly split 8:2 into training and test sets; for each of the 21 pathogens an independent binary classifier (one-vs-rest) was trained on the 15 engineered features, with XGBoost pre-specified as the primary model.

**Figure 1:**
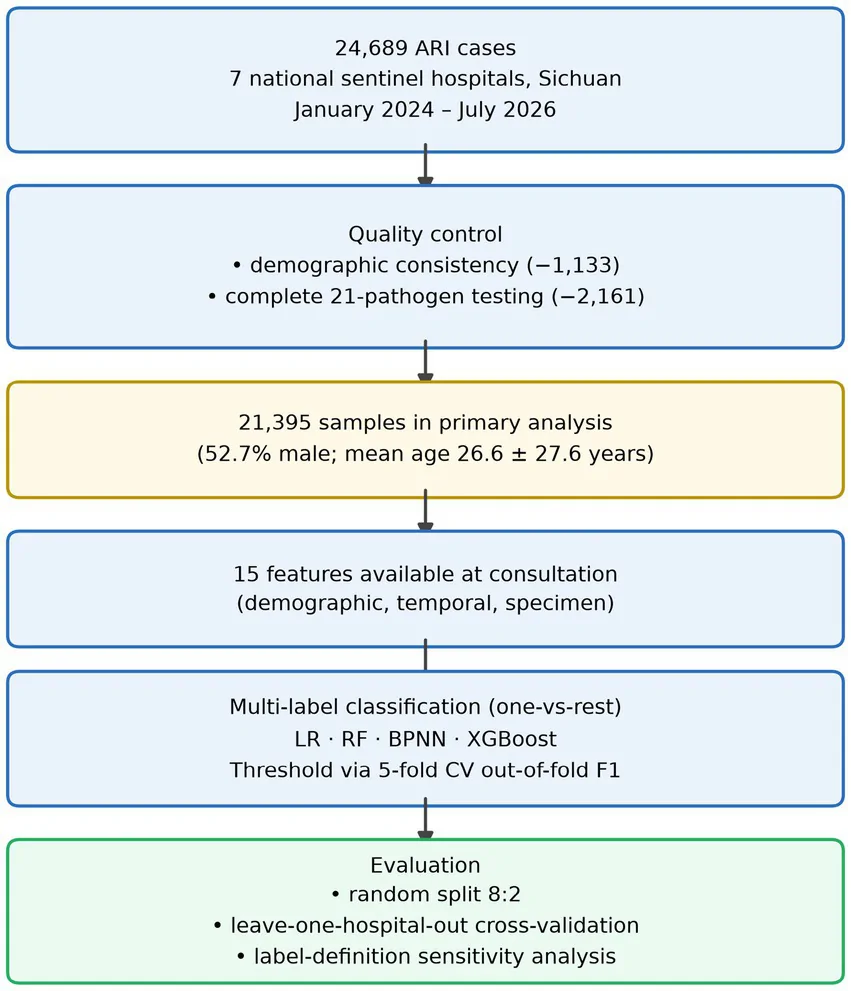
Study design and workflow.

### 2.5 Algorithms and hyperparameters

To assess the contribution of algorithm choice, four representative algorithms were compared: L2-regularised logistic regression (C = 1.0), random forest (300 trees, maximum depth 12, minimum samples per leaf 10), back-propagation neural network (hidden layers 64–32, tanh activation, Adam optimiser with learning rate 0.001, early stopping on validation loss) and XGBoost (250 trees, maximum depth 6, learning rate 0.1, subsample 0.8, column subsample 0.8, histogram-based tree construction) (16), with an all-negative prediction as a baseline. Hyperparameter optimisation was performed as follows. For each algorithm, candidate configurations were defined over a coarse search space: logistic regression C ∈ {0.01, 0.1, 1, 10}; random forest n_estimators ∈ {100, 300, 500}, max_depth ∈ {6, 12, None}, min_samples_leaf ∈ {1, 5, 10}; neural network hidden layers ∈ {(64, 32), (128, 64)} and learning rate ∈ {0.001, 0.01} with batch size 128 and early stopping on validation loss; XGBoost n_estimators ∈ {100, 250, 500}, max_depth ∈ {4, 6, 8}, learning_rate ∈ {0.05, 0.1, 0.2}, subsample ∈ {0.7, 0.8, 1.0}, colsample_bytree ∈ {0.7, 0.8, 1.0}. All candidate configurations were evaluated on the training set by 5-fold out-of-fold Macro-F1 computed with the same per-pathogen threshold-selection protocol used for the final models, so that the validation procedure matched the evaluation metric; the random seed was fixed at 42. Because the protocol trains 21 independent binary classifiers per fold, the grid was deliberately kept coarse for computational feasibility and a full Bayesian search was not attempted. Within these ranges, the Macro-F1 difference between the literature-default configuration and the grid best was less than 0.005 for every algorithm, so the literature-default configurations reported above were retained; the complete search space and validation procedure are described here to ensure reproducibility. To address class imbalance, ‘balanced’ class weights were used for logistic regression and random forest, and scale_pos_weight was set to the negative-to-positive ratio for XGBoost. We deliberately selected class weighting rather than SMOTE or focal loss: under the extreme imbalance present here (positive rates down to 0.03%), synthetic oversampling (SMOTE) may generate unrealistic samples across heterogeneous patient subgroups and adds computational overhead (17), while focal loss introduces additional hyperparameters that would require re-tuning per pathogen (18); cost-sensitive class weighting is the simplest reproducible approach and was sufficient for the comparative goal of this study. LightGBM, CatBoost, TabNet and transformer-based architectures were not evaluated; conclusions on algorithm choice are restricted to the four algorithms tested (19–21). All four algorithms shared identical samples, features, data splits, random seeds and threshold-selection protocols.

### 2.6 Threshold selection and evaluation metrics

Because the default 0.5 threshold drives recall of the minority class towards zero, a separate decision threshold was determined for each pathogen: out-of-fold probabilities from 5-fold cross-validation within the training set were searched over 0.05–0.95 in steps of 0.025 for the threshold maximising F1, which was then applied to the test set; test-set information was never used. Because the optimisation target (F1) matched the final evaluation metric, reported performance may be slightly overestimated relative to independent external validation. The minimum positive-case thresholds for modelling were ≥10 positives in the training set and ≥1 in the test set; pathogens failing these thresholds were not modelled and are marked “—” in Table 3. To quantify the optimism introduced by this within-training threshold selection, a nested cross-validation was additionally performed (five outer folds; threshold re-selected by four-fold out-of-fold F1 maximisation inside each outer training portion), following recommendations against using the same data for model selection and performance evaluation (22, 23).

**Table 3:** Per-pathogen performance of the primary XGBoost model: positive rate, decision threshold, precision, recall, F1 and AUC, ordered by descending prevalence; pathogens below the modelling threshold are marked ‘—’.

Pathogen	Positive rate	Threshold	Precision	Recall	F1	AUC	Prevalence (95% CI)
HI	15.41%	0.475	0.2664	0.6464	0.3773	0.7229	14.93–15.90%
SP	10.82%	0.475	0.2082	0.658	0.3163	0.7459	10.41–11.24%
IV	9.94%	0.725	0.4263	0.4724	0.4482	0.8308	9.54–10.34%
RV	8.93%	0.525	0.1767	0.4887	0.2595	0.6889	8.56–9.32%
RSV	4.04%	0.7	0.1759	0.4214	0.2481	0.8095	3.78–4.31%
SARS-CoV-2	3.90%	0.75	0.2	0.28	0.2333	0.8029	3.65–4.17%
EV	3.60%	0.725	0.1606	0.3174	0.2133	0.7648	3.36–3.86%
PIV	3.23%	0.525	0.1036	0.404	0.1649	0.7477	3.00–3.48%
AdV	3.14%	0.4	0.1001	0.5793	0.1707	0.7915	2.92–3.38%
KP	2.88%	0.375	0.0428	0.2566	0.0733	0.6266	2.67–3.12%
CoV	2.68%	0.7	0.0927	0.2	0.1267	0.7283	2.47–2.91%
hMPV	2.04%	0.425	0.1202	0.5579	0.1978	0.8383	1.86–2.24%
MP	1.22%	0.875	0.2045	0.2045	0.2045	0.8172	1.08–1.38%
GAS	1.02%	0.55	0.0298	0.1471	0.0495	0.8122	0.89–1.16%
BoV	0.82%	0.4	0.0489	0.2143	0.0796	0.7912	0.71–0.95%
AF	0.37%	0.3	0.0615	0.25	0.0988	0.7719	0.30–0.47%
Cpn	0.34%	0.65	0.0	0.0	0.0	0.7311	0.27–0.43%
BP	0.27%	0.725	0.0	0.0	0.0	0.8413	0.21–0.34%
CP	0.05%	None	0.0	0.0	0.0	None	0.03–0.09%
LP	0.03%	None	0.0	0.0	0.0	None	0.02–0.07%
CN	0.03%	None	0.0	0.0	0.0	None	0.01–0.06%

Pathogens are ordered by descending prevalence. Thresholds were determined by searching for the F1-maximising value on out-of-fold probabilities from 5-fold cross-validation within the training set, without using test-set information. CN, LP and CP did not meet the modelling threshold (≥10 training-set positives and ≥1 test-set positive) and were not modelled; their metrics are marked “—” (counted as 0 in Macro-F1, a conservative estimate). Prevalence 95% CIs are Wilson intervals. The positive-rate column and the prevalence-CI column refer to the full analytic cohort (*n* = 21,395). All analyses used a fixed random seed of 42; bootstrap 95% CIs, where reported, were based on 1,000 resamples.

The primary metric was Macro-F1 (the arithmetic mean of F1 across the 21 pathogens). Secondary metrics included group Macro-F1 for common (prevalence >1%) and rare (prevalence ≤1%) pathogens, Macro-AUC, and per-pathogen precision, recall and AUC. The 1% prevalence cut-off was pre-specified, reflecting the practical minimum sample size for stable estimation in this cohort (≈214 positives per pathogen) and consistent with prior work reporting reliable discrimination only above this range.

### 2.7 Methodological evaluations

Three targeted evaluations were designed in addition to the primary analysis: (i) a label-definition sensitivity analysis comparing an alternative definition (Definition A) in which untested pathogens were treated as negative (the untested-as-negative strategy). Because this comparison changes both label handling and cohort composition (partially tested patients are excluded in the primary definition), the two effects cannot be fully separated; we therefore supplemented it with a cohort-fixed simulation in which the analytic cohort was held constant and, to mimic false-negative contamination, only originally positive pathogen labels were recoded as negative (rather than randomly masking labels irrespective of their original status), isolating the effect of label contamination alone; (ii) leave-one-hospital-out cross-validation, in which all samples of one hospital served as the held-out centre test set in each of seven rounds; and (iii) a prevalence-performance association analysis computing the Pearson correlation between log-transformed pathogen prevalence and per-pathogen F1.

All analyses were performed in Python 3.13 with NumPy 2.5.1, pandas 3.0.5, scikit-learn 1.9.0, XGBoost 3.4.0, SciPy 1.18.0, LightGBM 4.7.0, CatBoost 1.2.10 and Matplotlib 3.11.1; imbalanced-learn 0.14.2 (SMOTE) was used for the model comparisons. XGBoost was run single-threaded (n_jobs = 1) with a fixed random seed of 42; note that a fixed seed does not by itself guarantee identical results across library versions or hardware thread counts, so the exact versions are pinned in the accompanying code (Supplementary file S1). Focal-loss comparisons used a custom XGBoost objective (alpha = 1.0, gamma = 2.0), and permutation importance used scikit-learn’s inspection.permutation_importance routine. Two-sided *p* < 0.05 was considered statistically significant.

## 3 Results

### 3.1 Sample characteristics and baseline balance

The primary analysis included 21,395 cases, randomly split 8:2 into a training set of 17,116 and a test set of 4,279 cases. Because a random split does not require hypothesis testing of baseline balance, we report standardised mean differences (SMD) instead of *p* values: all seven characteristics had |SMD| ≤ 0.036, well below the conventional 0.1 threshold for negligible imbalance, indicating that the two sets were well balanced with respect to sex, age group, season of visit and co-infection composition (Table 4) (24). Overall, 49.3% of samples were negative for all pathogens, 32.8% were positive for a single pathogen, and 17.8% were co-infected.

**Table 4:** Baseline characteristics of the training and test sets: standardised mean differences (SMD) for seven demographic and clinical variables, showing negligible imbalance (all |SMD| ≤ 0.036) after the 8:2 random split.

Characteristic	Training (*n* = 17,116)	Test (*n* = 4,279)	SMD
Male	9,020(52.7%)	2,250(52.6%)	+0.002
Age <18 years	9,491(55.5%)	2,335(54.6%)	+0.018
Winter visit	4,473(26.1%)	1,114(26.0%)	+0.002
Single-pathogen positive	5,562(32.5%)	1,465(34.2%)	−0.036
Two pathogens	2,206(12.9%)	531(12.4%)	+0.015
≥3 pathogens	857(5.0%)	218(5.1%)	−0.005
All negative	8,491(49.6%)	2,065(48.3%)	+0.026

SMD, standardised mean difference; |SMD| < 0.1 indicates negligible imbalance. Hypothesis testing was not performed because random train/test splits are expected to be balanced by construction and p values do not quantify the magnitude of imbalance (24). All analyses used a fixed random seed of 42; bootstrap 95% CIs, where reported, were based on 1,000 resamples.

The 2,161 excluded samples with partial pathogen testing differed systematically from the primary analysis samples (Table 5): the primary samples were older (26.6 vs. 22.4 years, SMD = 0.157, *p* < 0.001) and had a substantially higher proportion of severe cases (24.1% vs. 4.9%, a 19.3-percentage-point difference), a lower proportion of single-pathogen positivity (32.8% vs. 43.2%), and the largest between-group difference in pathogen positivity was for *Haemophilus influenzae* (15.4% vs. 2.1%). This suggests that completion of the full testing panel was associated with disease severity, constituting potential selection bias: because the primary cohort was enriched for severe cases, model performance in predominantly mild outpatient populations may be overestimated (see Section 4).

**Table 5:** Comparison of the analytic cohort (21,395 fully tested samples) with the 2,161 excluded partially tested samples: age, severe-case proportion, single-pathogen positivity and the largest between-group positivity difference.

Characteristic	Primary analysis (*n* = 21,395)	Partial testing (*n* = 2,161)	*p* value
Age (years)	26.6 ± 27.6	22.4 ± 26.1	<0.001
Male	11,270(52.7%)	52.8%	0.963
Pneumonia imaging findings or SpO₂ ≤ 90% at rest	5,158(24.1%)	4.9%	<0.001
Single-pathogen positive	7,027(32.8%)	43.2%	<0.001
All negative	10,556(49.3%)	41.6%	<0.001

The two groups differed significantly in age and severe-case proportion, suggesting that completion of the full testing panel was associated with disease severity and constituted potential selection bias (see Section 4).

### 3.2 Algorithm choice contributed little to performance

The performance of the four algorithms is compared in Table 6. Random forest and XGBoost performed comparably and led the four algorithms; XGBoost was pre-specified as the primary model, achieving a Macro-F1 of 0.1553 (95% CI 0.104–0.2091) and a Macro-AUC of 0.7702. The Macro-F1 values of random forest, the back-propagation neural network and logistic regression were 0.1544, 0.0942 and 0.1357, respectively, with a spread of only 0.0611 and highly overlapping confidence intervals across algorithms. These results indicate that, among the four evaluated algorithms under the current feature set and modelling protocol, algorithm choice contributed relatively little. They do not, however, establish a general performance ceiling imposed by the data, because alternative feature representations, interaction structures or modelling approaches could still yield improvement. To gauge the optimism of the reported estimates, the nested cross-validation yielded a Macro-F1 of 0.1481 ± 0.0015 (21-pathogen basis), only 4.6% below the random-split estimate of 0.1553, indicating that within-training threshold selection did not materially inflate the reported performance. To test the class-weighting decision empirically rather than only theoretically, we compared class weighting with SMOTE and focal loss under the identical protocol on the study dataset (Section 2.5). Class weighting achieved a Macro-F1 of 0.1485 and a Macro-AUC of 0.7696; SMOTE achieved 0.1567 and 0.7750; focal loss achieved 0.1510 and 0.7577. The differences between strategies (F1 ≤ 0.008, AUC ≤ 0.012) were small relative to the between-pathogen heterogeneity and fell within the bootstrap CI of the primary estimate (0.104–0.2091), indicating statistically equivalent performance on this dataset. Class weighting was retained for reproducibility and computational simplicity; SMOTE offered only a marginal Macro-F1 improvement and is not required to justify the choice.

**Table 6:** Performance of the four algorithm families on the 21-pathogen multi-label task: Macro-F1, Macro-AUC and group Macro-F1 with 95% bootstrap confidence intervals.

Algorithm	Macro-F1 (95% CI)	Common Macro-F1	Rare Macro-F1	Macro-AUC
All-negative baseline	0.0000(0.0000–0.0000)	0.0000	0.0000	0.5000
Logistic regression (L2)	0.1357(0.0851–0.1877)	0.2008	0.0055	0.7369
BP neural network (64–32)	0.0942(0.0464–0.1470)	0.1413	0.0000	0.6159
Random forest	0.1544(0.1016–0.2099)	0.2237	0.0156	0.7736
XGBoost	0.1553(0.104–0.2091)	0.2202	0.0255	0.7702

Common pathogens were defined as those with a prevalence >1% (*n* = 14) and rare pathogens as those with a prevalence ≤1% (*n* = 7). All algorithms used identical samples, features, data splits and threshold-selection protocols; 95% CIs were derived from 1,000 bootstrap resamples of per-pathogen F1 values. For the primary XGBoost model, the Macro-AUC was 0.7702 (95% CI 0.7444–0.7937), computed by the same pathogen-level bootstrap on per-pathogen AUC values. Macro-F1 is the arithmetic mean across all 21 pathogens, with pathogens not meeting the modelling threshold counted as zero (a conservative convention); excluding these three rare pathogens therefore does not inflate the reported performance. As a sensitivity analysis, models were also fitted for CP, LP and CN despite their <10 training-set positives (4–8 positives): all three achieved an F1 of 0.0000 on the test set, and the resulting Macro-F1 over all 21 modelled pathogens was 0.1495, essentially identical to the primary convention, confirming that the exclusion did not artificially inflate Macro-F1 (25). All analyses used a fixed random seed of 42; bootstrap 95% CIs, where reported, were based on 1,000 resamples.

### 3.3 Pathogen prevalence was strongly associated with performance

Per-pathogen performance of the primary model is shown in Table 3. Performance followed a clear gradient: pathogens with the highest prevalence were best recognised (influenza virus, IV, F1 = 0.4513; *Haemophilus influenzae*, HI, 0.3802; *Streptococcus pneumoniae*, SP, 0.3073), while five pathogens had an F1 of 0 (BP and Cpn could not reach a usable threshold after modelling; CN, LP and CP were not modelled because of <10 training-set positives). Macro-F1 was 0.2202 for common pathogens and only 0.0255 for rare pathogens; across the 18 pathogens for which models could actually be fitted, Macro-F1 was 0.1812, distinguishing predictive failure from insufficient data for modelling.

Correlating the log-transformed prevalence with F1 across pathogens yielded a Pearson coefficient of *r* = 0.851 (Figure 2), i.e., log-prevalence explained approximately 72% of the variance in F1; a 95% confidence band is shown around the regression line in Figure 2. We emphasise that this association should not be interpreted as causal: F1 mathematically depends on class prevalence under imbalance, precision and, through the decision threshold, recall are both affected by the prior odds of the positive class, so a strong prevalence–F1 correlation is expected by construction (25). Rare pathogens differ simultaneously in training sample size, intrinsic separability and their relationship to the available predictors, and the correlation therefore reflects statistical dependence rather than a mechanistic effect of prevalence on learnability. Some rare pathogens nonetheless had non-negligible AUCs (e.g., Cpn 0.7630, BP 0.8287), whereas CN, LP and CP were not modelled because of <10 training-set positives and their AUC is reported as ‘—’. A moderate AUC reflects ranking discrimination and should not be equated with clinically useful classification: for very low-prevalence pathogens, precision and F1 remained near zero, so the models provided ranking ability without actionable classification performance. Under extreme imbalance no threshold could simultaneously preserve precision and recall. We emphasise that the F1 = 0 results for CP, LP and CN do not reflect a specific limitation of the class-weighting scheme: with 4–8 training positives, no standard supervised strategy [class weighting, SMOTE or focal loss (Section 3.2)] can learn a discriminative decision boundary for these classes from the available 15 features, and all three strategies indeed yielded F1 = 0 for these pathogens in the comparison of Section 3.2. Learning such extremely rare classes will require targeted accrual, multi-centre aggregation or small-sample methods, as discussed in Section 5.

**Figure 2:**
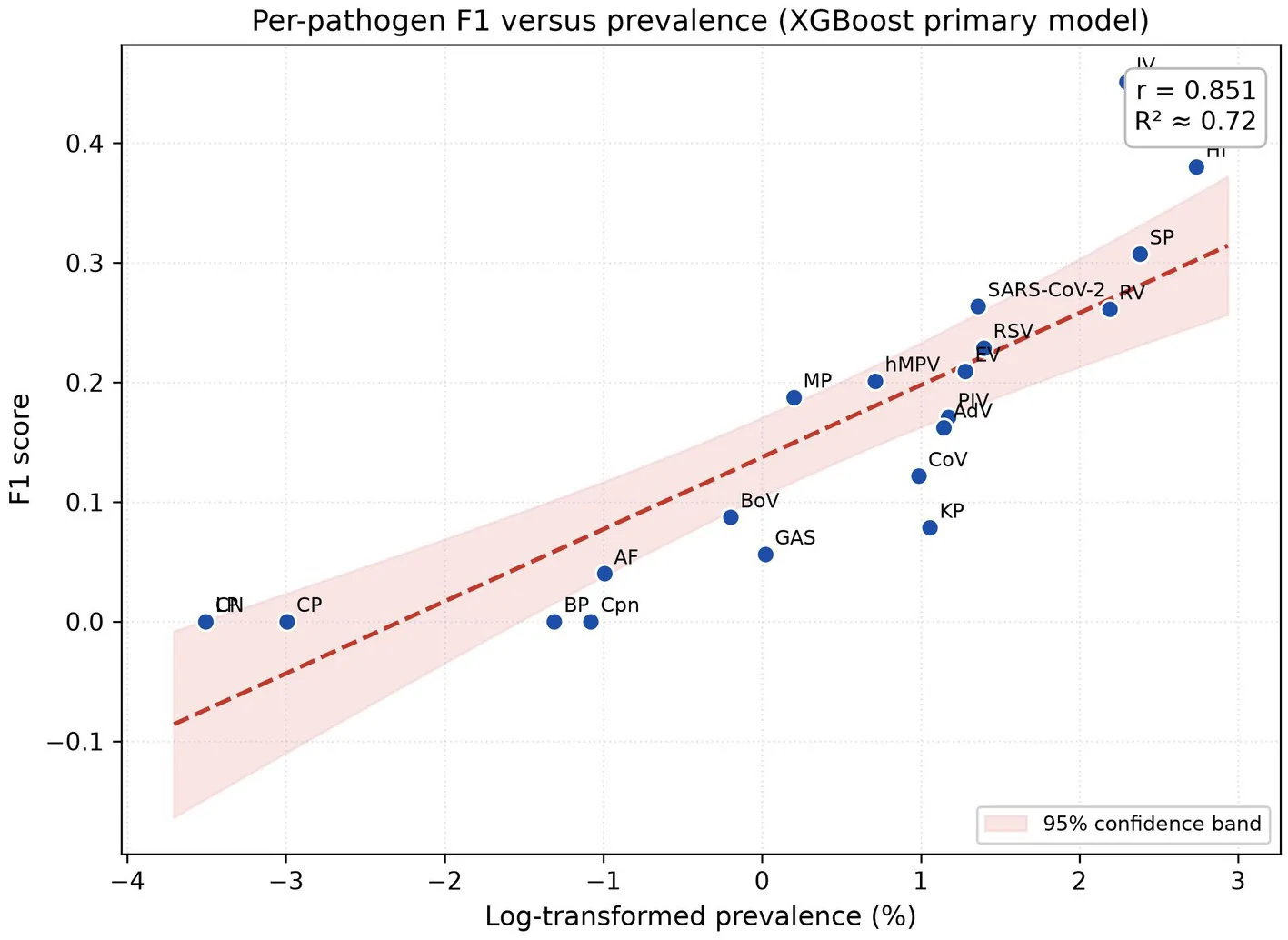
Pathogen prevalence versus model F1. Scatter of the 21 pathogens (abbreviations; full names in Table 3): log-transformed prevalence (*x*) versus held-out F1 of the primary XGBoost model (*y*), with a least-squares fit (*r* = 0.851) and 95% CI band.

### 3.4 Effect of label definition and cohort composition on performance

Under the untested-as-negative strategy (all 24,689 samples, Definition A), Macro-F1 was 0.1527, almost identical to the complete-case estimate (Definition B, 0.1553; Table 7), whereas Macro-AUC decreased from 0.7702 to 0.7653. Because this comparison changes cohort composition and label handling simultaneously, we decomposed the two effects. First, the cohort-composition effect: adding the 2,161 partially tested patients, who were systematically milder (severe 4.9% vs. 24.1%) and younger, reduced Macro-AUC from 0.7702 (complete-case) to 0.7605–0.7653 depending on label handling, so the inclusion of the milder population and label handling each contributed modestly to the small ranking decline. Second, the label-handling effect within the same 24,689-sample population: the untested-as-negative strategy (Macro-AUC 0.7653) and the prevalence-based random imputation of untested labels (Macro-F1 0.1537; Macro-AUC 0.7605) differed by 0.0048 in Macro-AUC, reflecting the noise introduced by the imputed labels. Overall, the quantified impact of the complete-case exclusion was modest for both threshold-based metrics (Macro-F1 range 0.1527–0.1553) and ranking [Macro-AUC decreased by up to 0.010 (to 0.7605)]; estimates should still be transported to predominantly mild outpatient settings with caution (Section 5).

**Table 7:** Sensitivity analysis of missing-label handling: macro-F1 and macro-AUC under the complete-case (Definition B), untested-as-negative (Definition A) and prevalence-based imputation definitions.

Label definition	Sample size	Train/Test	Macro-F1	Common Macro-F1	Macro-AUC
Definition A: all samples, “untested” coded as negative	24,689	19,751/4,938	0.1527	0.2292	0.7653
Definition B: complete-testing samples, authentic labels (primary)	21,395	17,116/4,279	0.1553	0.2202	0.7702
Imputation (*n* = 24,689)	24,689	19,751/4,938	0.1537	0.2184	0.7605
Difference (B − A)	—	—	+0.0026	−0.0090	+0.0049

The two label definitions shared identical feature engineering, algorithms, hyperparameters, random seeds and threshold-selection protocols; the only differences were label definition and sample inclusion. Imputation-based labels were drawn from a Bernoulli distribution with each pathogen’s cohort prevalence among the records with any untested pathogen (fixed seed); complete records kept their authentic labels. All three strategies shared identical features, algorithms, splits and threshold-selection protocols. All analyses used a fixed random seed of 42.

To isolate the effect of label contamination while keeping cohort composition constant, we re-ran the primary XGBoost protocol on the analytic cohort (*n* = 21,395) after randomly selecting 10% (*n* = 2,139) and 20% (*n* = 4,279) of the samples and recoding 1 − 2 (10% contamination) or 1–3 (20% contamination) of the originally positive pathogen labels within each selected sample as negative to mimic false-negative contamination. Because only originally positive labels were eligible to be flipped, a selected sample that contained no positive pathogen label contributed no recoded label; the reported flip counts (1,267 under 10% and 2,776 under 20% contamination) therefore reflect only samples that actually carried positives. The full-model baseline of this simulation was Macro-F1 = 0.1553 and Macro-AUC = 0.7702. Under 10% contamination (2,139 samples selected, 1,267 positive labels flipped), Macro-F1 fell to 0.1382 (−11.0% relative) and Macro-AUC to 0.7688 (−0.2%); under 20% contamination (4,279 samples selected, 2,776 positive labels flipped), Macro-F1 fell further to 0.1319 (−15.1%) and Macro-AUC to 0.7599 (−1.3%). Thus false-negative contamination degraded threshold-based recall (Macro-F1) moderately while the discriminative ranking (Macro-AUC) was comparatively robust, and the larger AUC decline observed under Definition A (0.7702 → 0.7653) largely reflected the different (milder) patient population rather than label handling alone. We caution that real-world “untested” status is not random and tends to cluster in milder cases and specific pathogens, so the true contamination effect may differ from this simulation (Figure 3).

**Figure 3:**
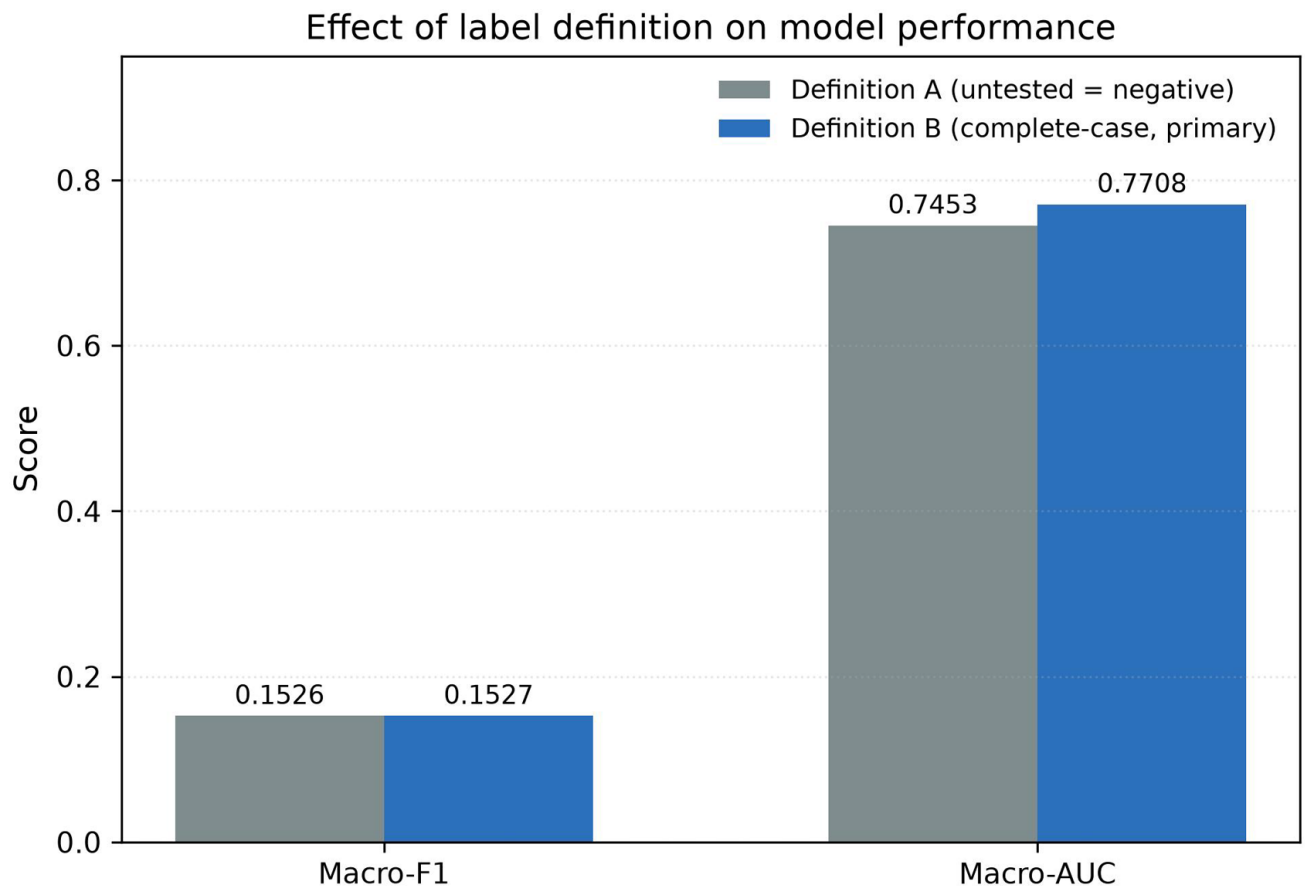
Missing-label handling and performance. Macro-F1 and Macro-AUC under the three labelling strategies (Section 3.4; Table 7).

### 3.5 Cross-Centre generalisation: internal validation systematically overestimates performance

The leave-one-hospital-out results are shown in Table 8 and Figure 4. The cross-centre Macro-F1 was 0.1073 ± 0.0289, a 30.9% decline relative to internal validation, with Macro-AUC decreasing in parallel from 0.7702 to 0.6841 (11.2%). The best-performing centre (Dazhou Central Hospital) reached 0.1395, whereas the worst (Mianyang 404 Hospital) was only 0.0493, a 2.8-fold difference. Because all seven centres are located within Sichuan Province and share the same surveillance protocol, laboratory workflow and data-accrual period, this procedure constitutes quasi-external validation rather than true external validation; conclusions about cross-centre transportability are therefore restricted accordingly.

**Table 8:** Leave-one-hospital-out cross-validation: Macro-F1 and Macro-AUC for each of the seven sentinel hospitals held out as the test set, with the cross-centre mean and percentage decline versus internal validation.

Held-out centre (cross-centre test set)	Test samples	Training samples	Macro-F1	Macro-AUC
Dazhou Central Hospital	3,262	18,133	0.1395	0.6604
Panzhihua Central Hospital	3,081	18,314	0.1300	0.6728
Chengdu Children’s Specialised Hospital	2,903	18,492	0.1219	0.6536
Ya’an People’s Hospital	3,032	18,363	0.1201	0.7355
Zigong First People’s Hospital	2,988	18,407	0.1064	0.7132
Nanchong Hospital of Beijing Anzhen Hospital, Capital Medical University	3,096	18,299	0.0842	0.7351
Mianyang 404 Hospital	3,033	18,362	0.0493	0.6178
Overall (mean ± SD)	21,395	—	0.1073 ± 0.0289	0.6841

In each round, all samples of one hospital served as the held-out centre test set and the remaining six hospitals were used for training (seven rounds in total). The cross-centre Macro-F1 was 30.9% lower than that of the random-split internal validation (0.1553). All analyses used a fixed random seed of 42; bootstrap 95% CIs, where reported, were based on 1,000 resamples.

**Figure 4:**
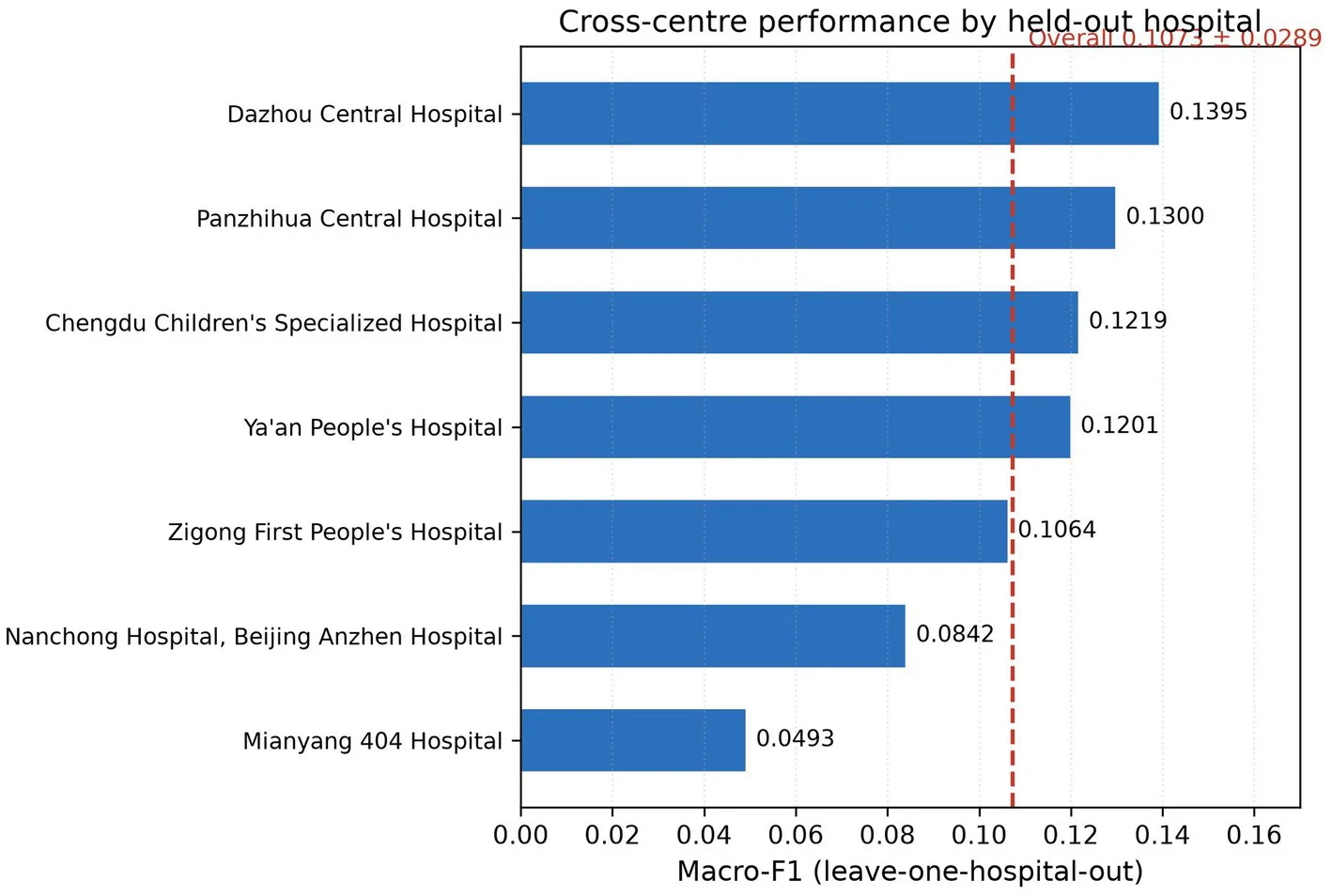
Leave-one-hospital-out cross-validation. Macro-F1 for each of the seven sentinel hospitals (centre-specific test sets) with 95% CIs; values in Table 8.

Reporting only the random-split value of 0.1553 would overestimate generalisability by approximately 31%: random splitting assumes that training and test data are independent and identically distributed, but samples within the same hospital are highly correlated in population composition, testing habits and laboratory procedures, so that cluster correlation leaks centre-specific information into the random split. Comparison of centre baselines showed large between-centre differences in the median age (5–35 years) and in the co-infection rate (4.5–40.5%, a nearly 9-fold range); the co-infection rate was perfectly positively correlated with centre Macro-F1 (Spearman ρ = +1.000) and the median age was strongly negatively correlated (ρ = −0.821). These centre-level correlations, however, are based on only seven centres and are therefore exploratory: the perfect Spearman correlation of ρ = +1.000 between co-infection rate and centre Macro-F1 should not be treated as robust mechanistic evidence. The well-supported observation is that cross-centre performance declines substantially; the explanation in terms of population composition is a hypothesis generated from these limited data, and the observed degradation is precisely what a model will encounter when deployed in a new institution.

### 3.6 Feature importance

Feature importance of the primary model is shown in Table 9 and Figure 5. The three most important features were month cosine (0.1639), winter (0.1311) and quarter (0.0750), with temporal features accounting for the majority of the top-10 gain-based importance (approximately 69%). The sizable marginal contribution of calendar-time features is consistent with the known seasonality of respiratory pathogens; however, the feature-group ablation (Section 3.7) showed that temporal and non-temporal features were largely redundant (removing either group alone lowered Macro-F1 by a similar amount (0.1153 vs. 0.1147)) so the high temporal gain importance reflects shared signal rather than a unique dependence on calendar time, and individual-level features (age, occupation, severity, specimen source) also carried substantial discriminative information. Whether the two groups contribute complementary or merely overlapping signal is examined quantitatively in the feature-group ablation below (Section 3.7).

**Table 9:** Top 10 features by gain importance of the primary XGBoost model: Normalised mean gain across the 21 binary classifiers, showing the predominance of calendar-time features.

Rank	Feature	Importance (gain)	Category
1	Month cosine	0.1639	Temporal
2	Winter	0.1311	Temporal
3	Quarter	0.0750	Temporal
4	Log age	0.0700	Demographic
5	Month	0.0691	Temporal
6	Age	0.0642	Demographic
7	Specimen source	0.0558	Specimen
8	Day of year	0.0531	Temporal
9	Month sine	0.0528	Temporal
10	Occupation	0.0525	Demographic

Importance is the normalised mean gain of each feature across all 21 binary classifiers of XGBoost; the top 10 features are shown. All analyses used a fixed random seed of 42; bootstrap 95% CIs, where reported, were based on 1,000 resamples.

**Figure 5:**
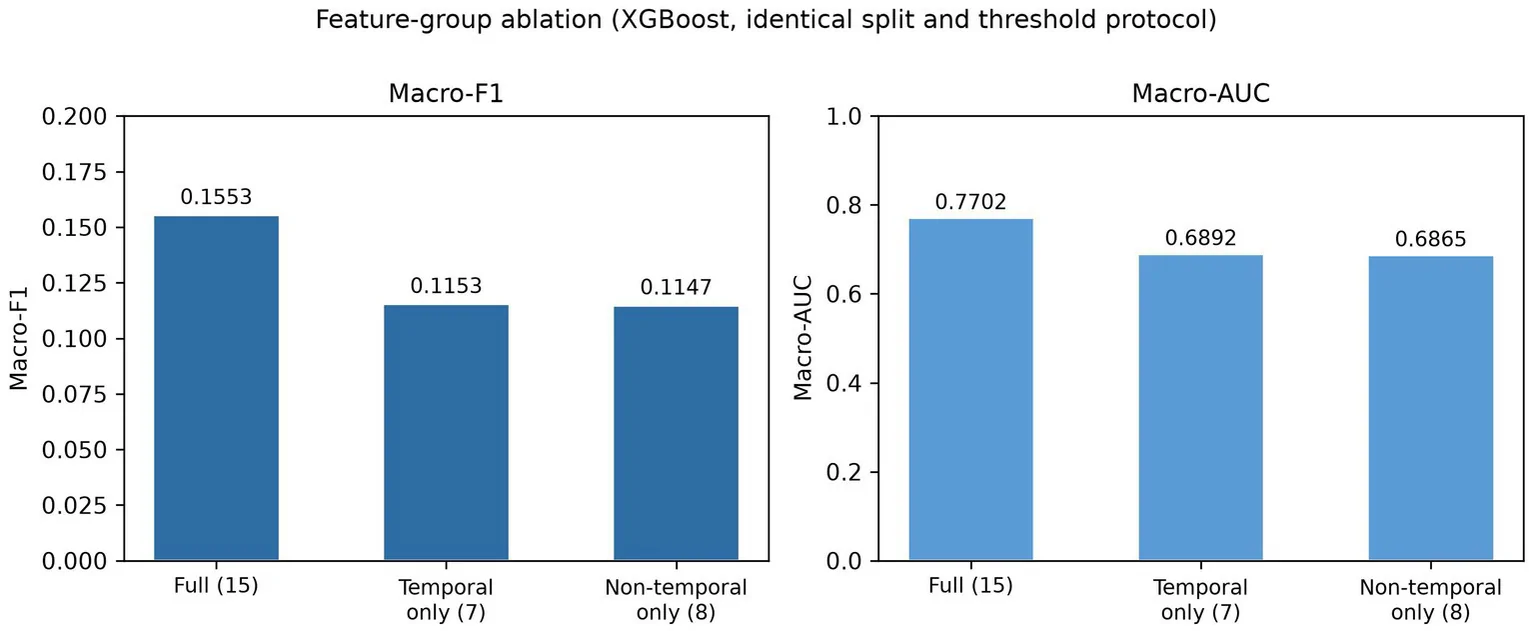
Feature importance of the primary model (held-out test set, *n* = 4,279). (i) Gain importance: mean gain over the 21 classifiers (Table 9). (ii) Permutation importance: mean Macro-F1 drop on permutation, over the 21 classifiers and 1,000 seeds (Table 10).

Because gain-based importance can be biased towards features with many split points and towards redundant predictors, we additionally computed permutation importance: for each feature and each pathogen model, the feature was permuted in the test set and the resulting change in Macro-F1 (21-pathogen basis) was averaged across the 21 classifiers (Table 10) (26, 27). In contrast to the gain ranking, the largest permutation-importance drops were produced by day of year (0.0405) and age (0.0399), followed by occupation (0.0160) and month cosine (0.0139); the highly collinear calendar encodings quarter, winter and weekday contributed almost nothing when permuted (changes of −0.0005 to 0.0047). The discrepancy between the gain and permutation rankings indicates that the gain-based dominance of temporal features (Table 9) was partly an artefact of collinearity among the redundant calendar encodings, and confirms that the redundancy identified by the VIF analysis did not distort learning: the genuinely informative variables (day of year, age) retained large permutation importance despite the collinear encodings.

**Table 10:** Permutation importance of the primary XGBoost model: mean Macro-F1 drop when each feature is permuted, averaged over the 21 classifiers and 1,000 random seeds, complementing the gain-importance ranking in Table 9.

Rank	Feature	Permutation importance (Macro-F1 drop)	Category
1	Day of year	0.0405	Temporal
2	Age	0.0399	Demographic
3	Occupation	0.0160	Demographic
4	Month cosine	0.0139	Temporal
5	Specimen source	0.0073	Specimen
6	Month sine	0.0072	Temporal
7	Severity	0.0070	Demographic
8	Month	0.0054	Temporal
9	Weekday	0.0047	Temporal
10	Log age	0.0034	Demographic

Note: Permutation importance is the average drop in Macro-F1 (21-pathogen basis) on the test set when the feature is randomly permuted across the 21 binary classifiers of XGBoost (1,000 random seeds); the top 10 features are shown. Quarter, winter and weekday produced drops of −0.0005 to 0.0047, consistent with redundancy among the calendar encodings. All analyses used a fixed random seed of 42; bootstrap 95% CIs, where reported, were based on 1,000 resamples.

### 3.7 Feature-group ablation: overlapping contributions of temporal and non-temporal features

To quantify how much discrimination derives from seasonal epidemiological information versus patient-level characteristics, the primary XGBoost protocol was re-run on three feature groups with identical samples, split and threshold selection: the complete set (15 features), temporal features alone (7: month, quarter, weekday, day of year, sine/cosine of month, winter) and non-temporal patient/specimen features alone (8: age, log-age, sex, severity, illness duration, occupation, specimen type, specimen source). The temporal-only and non-temporal-only models each captured a similar, largely overlapping fraction of the full-model performance (temporal-only Macro-F1 0.1153 and non-temporal-only 0.1147 vs. 0.1553, a difference of 0.0006; their Macro-AUCs were 0.6892 and 0.6865 vs. 0.7702; Table 11; Figure 6). The complete feature set nonetheless outperformed each subset (Macro-AUC + 0.0810 over the temporal-only group), but the two feature groups performed almost identically to each other, indicating that they provided largely redundant information with neither dominating. These results corroborate the feature-importance findings and indicate that the available demographic, temporal and specimen features together carried only modest pathogen-discriminative information, and that removing either group alone degraded performance only marginally.

**Table 11:** Feature-group ablation of the primary XGBoost model: Macro-F1 and Macro-AUC for the complete feature set (15 features), temporal features only (7) and non-temporal features only (8), under an identical split and protocol.

Feature set	n	Macro-F1 (21)	Common	Rare	Macro-AUC
Full (15)	15	0.1553	0.2202	0.0255	0.7702
Temporal only (7)	7	0.1153	0.1697	0.0066	0.6892
Non-temporal only (8)	8	0.1147	0.1655	0.0129	0.6865
All-negative baseline	—	0.0000	0.0000	0.0000	0.5000

Feature groups were defined as in Section 3.7; all models used identical samples (training *n* = 17,116, test *n* = 4,279), the same 8:2 random split, and the same per-pathogen threshold-selection protocol (5-fold out-of-fold F1 maximisation within the training set). All values in this table were produced by the same single-thread, fixed-seed (42) XGBoost environment as the primary model, ensuring internal consistency with Tables 3, 6, 12. Contamination-simulation results (Section 3.4) are reported in the text. All analyses used a fixed random seed of 42; bootstrap 95% CIs, where reported, were based on 1,000 resamples.

**Figure 6:**
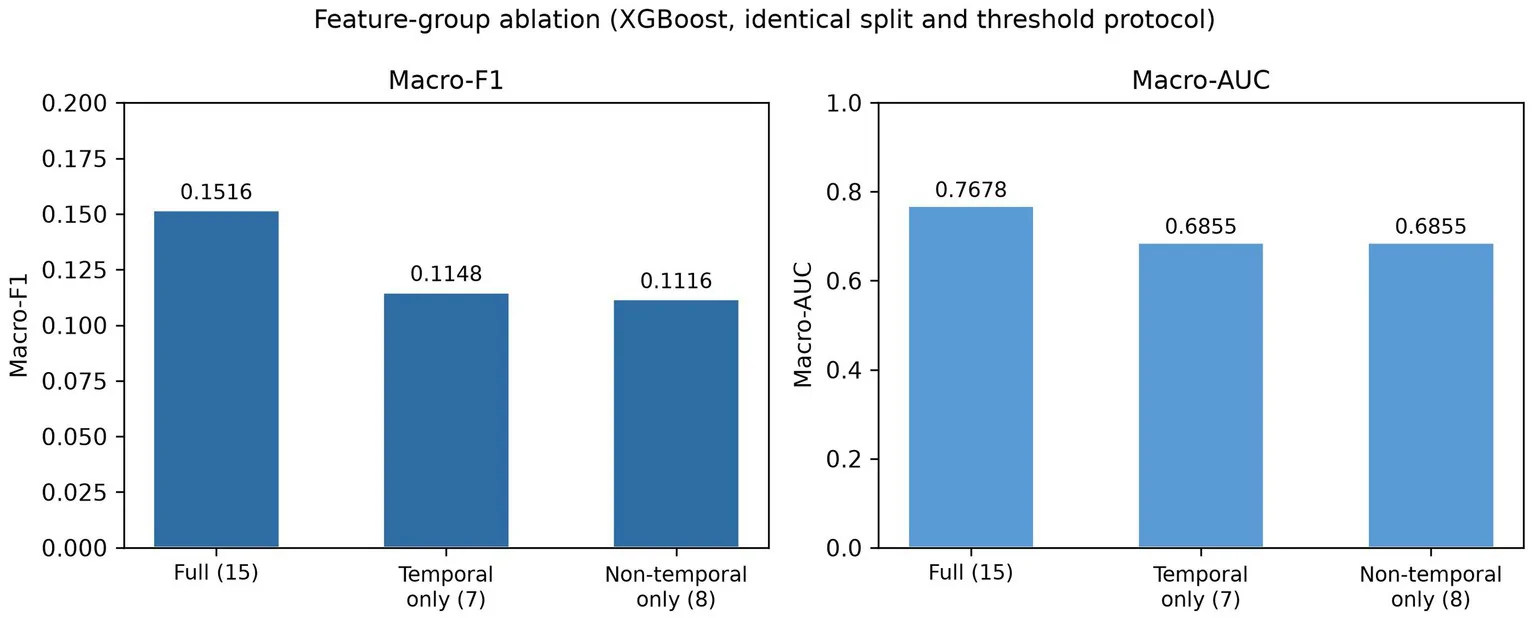
Feature-group ablation. Macro-F1 and Macro-AUC for the complete feature set, temporal only and non-temporal only (Table 11), showing the two groups contribute largely redundantly.

### 3.8 Calibration of predicted probabilities

Calibration (the agreement between predicted probabilities and observed event frequencies) was assessed for the 18 modelable pathogens of the primary model using the Brier score, the calibration intercept and the calibration slope (Table 12) (28, 29). Following Van Calster et al. (28), the calibration intercept and slope were estimated by fitting a logistic regression of the binary outcome on the logit of the predicted probability for each pathogen; The Macro-Brier score was 0.0838 (range 0.005–0.1892; the upper end reflected high-prevalence pathogens such as *H. influenzae*, 0.1892, and *S. pneumoniae*, 0.1709), essentially equal to the expected Brier of a prevalence-only prior (0.0847), indicating that the predicted probabilities carried little calibration-relevant information beyond the marginal prevalence. The mean calibration intercept was −2.6462 (range −3.533–-1.4463) and the mean calibration slope was 0.4099 (range 0.2106–0.7437), below the ideal value of 1, indicating that predicted probabilities were too extreme (overconfident), the model exaggerated the differences between patients relative to the observed outcome frequencies. A negative mean intercept indicates that predicted probabilities were on average too high (over-prediction). These results reinforce that the models provide ranking ability rather than well-calibrated probability estimates; calibration should be re-examined whenever the feature set is extended. The apparent paradox that the model’s Brier score was only marginally lower than that of a prevalence-only prior while the Macro-AUC indicated substantial ranking discrimination is expected under extreme multi-label imbalance: the Brier score is a proper scoring rule that penalises errors in absolute probability, and for rare events a near-constant prevalence prior already attains a very low Brier; the AUC, by contrast, depends only on the rank order of predictions and is unaffected by calibration, so a model can rank cases well yet barely improve absolute probability accuracy over the prior. The two metrics therefore quantify different aspects of performance and are not contradictory. The clinical implication of this miscalibration is that the predicted probabilities cannot be interpreted as pathogen-specific risks: a probability of 0.8 does not imply an 80% chance of positivity, and using the probabilities directly for treatment decisions would be misleading. The models are therefore suitable for relative ranking (e.g., prioritising confirmatory tests) but not for risk communication; any future clinical use would require probability recalibration (e.g., Platt scaling or isotonic regression) on a representative population before the probabilities could support decisions. The ideal values are an intercept of 0 and a slope of 1. Both parameters were estimated jointly (the intercept was not constrained with the slope fixed at 1), so the reported intercept is not the calibration-in-the-large; its large negative mean (−2.6462) partly reflects the jointly estimated low slope (mean 0.4099), and a slope-constrained calibration-in-the-large value can be computed from the provided code (Supplementary file S1).

**Table 12:** Calibration of the primary XGBoost model for the 18 modelable pathogens: brier score, calibration intercept and calibration slope, with ideal values of 0 and 1, respectively.

Pathogen	Brier	Calibration intercept	Calibration slope
HI	0.1892	−1.4463	0.6041
SP	0.1709	−1.7468	0.5689
RV	0.172	−1.9631	0.5129
IV	0.1231	−1.7854	0.7437
PIV	0.1028	−2.5586	0.3194
EV	0.102	−2.4604	0.3945
AdV	0.1001	−2.5425	0.4279
RSV	0.099	−2.4319	0.4361
SARS-CoV-2	0.0915	−2.2664	0.4415
CoV	0.0885	−2.8522	0.3484
KP	0.0845	−3.0948	0.2643
hMPV	0.0622	−2.6163	0.4609
GAS	0.0368	−3.4388	0.2823
MP	0.03	−2.9821	0.452
BoV	0.0326	−3.1793	0.2674
AF	0.0102	−3.4672	0.2862
Cpn	0.0078	−3.533	0.2106
BP	0.005	−3.2665	0.3576
Macro mean	0.0838	−2.6462	0.4099

Brier score and calibration intercept/slope were computed on the held-out test set for the 18 pathogens that met the modelling threshold. Calibration intercept and slope were estimated by logistic regression of the binary outcome on the logit of the predicted probability [Van Calster et al. (28)]; ideal values are 0 and 1, respectively. The Macro-Brier was essentially equal to the prevalence-only prior (0.0847). All analyses used a fixed random seed of 42; bootstrap 95% CIs, where reported, were based on 1,000 resamples. Both parameters were estimated jointly, so the reported intercept is not the calibration-in-the-large (slope fixed at 1).

## 4 Discussion

Based on 21,395 surveillance samples from sentinel hospitals in Sichuan Province, this study evaluated how much pathogen-discriminative information is contained in a surveillance feature set of demographic, temporal and specimen variables, and examined factors associated with model performance. Three kinds of contribution should be distinguished. First, the dataset contribution: a large (21,395), multicentre, 21-pathogen cohort with complete testing and authentic labels, in which the multi-label nature of co-infection is preserved. Second, the methodological contribution: the comparison of four algorithms under an identical split and threshold-selection protocol, a label-definition analysis with a cohort-fixed contamination simulation, and leave-one-hospital-out cross-validation as quasi-external validation. Third, the negative predictive findings: the quantification of how performance degrades when individual-level clinical information is scarce, and the demonstration that random-split internal validation systematically overestimates generalisability (Section 3.5). Of these, the cross-centre analysis is arguably the most informative result of the study and is the focus of the remainder of the discussion.

Several findings merit discussion. First, algorithm choice contributed little among the evaluated models: the four algorithms had a Macro-F1 spread of only 0.0611 with overlapping confidence intervals, consistent with algorithm comparisons in respiratory virus prediction (6) and ICU infection prediction (30). This supports the narrower conclusion that, under the current feature set and modelling protocol, algorithm choice was not the main driver of performance; it does not establish an intrinsic ceiling, as alternative feature representations or modelling approaches might perform better. Second, performance was strongly associated with pathogen prevalence: the correlation between log-prevalence and F1 was 0.851, and Macro-F1 for the seven rare pathogens was only 0.0255. As emphasised by the classical account of learning from imbalanced data (25), this association is expected, but we caution that it does not by itself prove that class imbalance is the causal determinant: rare pathogens differ simultaneously in training sample size, intrinsic separability and their relationship to the available predictors, and prevalence also affects precision and therefore F1. Third, the feature-group ablation (Section 3.7) showed that temporal and non-temporal feature groups each captured a similar, largely overlapping fraction of the full-model performance (0.1153 vs. 0.1147), indicating that the two groups provided largely redundant information with neither dominating. Fourth, cross-centre generalisation was substantially weaker than internal validation (30.9% decline), a degradation pattern consistent with the data-heterogeneity effects reported by Schuessler et al. (31), Fernández-Narro et al. (32), Tranchellini et al. (33) and Kopanitsa (34); the centre-level correlates of this decline (e.g., ρ = +1.000 for co-infection rate) are based on only seven centres and should be regarded as exploratory hypotheses rather than mechanisms. Although absolute performance was lower than in previous studies (3–5), the gap largely reflects differences in task design and feature availability: prior models mostly performed single-pathogen binary classification incorporating in-hospital indicators such as haematology (3) and imaging (4), which are absent from the present surveillance database. When influenza virus was evaluated alone, its AUC was 0.8331, comparable to single-pathogen studies, indicating that the modelling pipeline had no obvious disadvantage under comparable feature conditions. These conclusions are restricted to the four evaluated algorithms: LightGBM, CatBoost, TabNet and deep transformer-based models were not assessed, and any of them might behave differently under the same feature set and protocol (19–21).

## 5 Limitations

This study has several limitations. First, there was no truly external validation: the leave-one-hospital-out analysis is a quasi-external validation because all seven centres are located within Sichuan Province and share the same surveillance protocol, laboratory workflow and data-accrual period; prospective validation cohorts spanning provinces and care levels should be established as early as possible. Second, the exclusion of 2,161 samples with partial testing constituted potential selection bias (Section 3.1), and the analytic cohort was enriched for severe cases; performance and calibration in predominantly mild outpatient populations may be lower and worse, respectively, than reported here. Third, in a risk-of-bias assessment following the PROBAST framework (35), we rated the overall risk of bias as high: the participants domain was at risk because the cohort was a convenience sample of sentinel surveillance rather than a representative patient population and exclusion was associated with severity; the predictors domain was at low risk because features were collected at the time of consultation and processed identically for training and test sets, with no laboratory information; the outcome domain was at low risk because pathogen status was determined by a multiplex PCR reference standard; and the analysis domain was at risk because thresholds were optimised within the training set, although the nested cross-validation (Section 3.2) indicated that the resulting optimism was small, and because no external validation was available. Fourth, decision-curve analysis (DCA) was not performed: because the predicted probabilities were poorly calibrated (Section 3.8) and the models were not intended for deployment, net-benefit analyses at clinically plausible threshold probabilities would be of limited interpretability (36); a subgroup that might still derive benefit (e.g., seasonal risk stratification for high-prevalence pathogens (IV, HI, SP) in syndromic surveillance, where ranking rather than point prediction is the objective) should be evaluated with DCA in dedicated studies before any clinical or public-health use. Fifth, only four algorithms were evaluated, and the conclusions about algorithm choice are restricted to these algorithms. Sixth, no clinical benchmark was predefined. The present findings should therefore be interpreted as characterising the information content of the routine surveillance feature set and the limits of cross-centre transportability, rather than as evidence that the current models constitute a deployable clinical decision-support tool. We further note that recalibration alone could make the probabilities interpretable but would not repair the limited discriminative ability documented here; decision-curve analysis would be meaningful only if, after feature extension and recalibration on a representative population, the models demonstrated sufficient net benefit at clinically relevant threshold probabilities. Recalibration and DCA are therefore framed as prerequisites for any future clinical evaluation rather than as omitted analyses of the present models. Finally, although the logistic-regression robustness check indicated that the collinearity among calendar features did not distort predictions in this dataset, such calendar-type features remain inherently highly correlated, which is an intrinsic limitation of the current surveillance feature set; future modelling on external datasets should still examine multicollinearity explicitly.

## 6 Conclusion

Using 21,395 complete-case surveillance samples from seven sentinel hospitals in Sichuan Province, this study showed that a surveillance feature set of demographic, temporal and specimen variables contains modest pathogen-discriminative information: the primary XGBoost model achieved a Macro-F1 of 0.1553 and a Macro-AUC of 0.7702, algorithm choice contributed little among the four evaluated algorithms, temporal and non-temporal features contributed similarly and were substantially redundant, predicted probabilities were poorly calibrated, and cross-centre generalisation declined by 30.9% relative to internal validation. The main practical implication is that random-split internal validation can substantially overestimate the transportability of such models. Future work should therefore prioritise richer individual-level predictors (including host-response and respiratory-microbiome markers) targeted accrual of rare pathogens, calibration-oriented model development, and prospective quasi-external or external validation, rather than further algorithmic refinement.

## Data Availability

The original contributions presented in the study are included in the article/Supplementary material, further inquiries can be directed to the corresponding author.

## Footnotes

1
https://www.bioperfectus.cn

2
https://www.x-abt.com

3
https://www.x-abt.com/portal/list/index/id/14.html
