# Correction: Pathogen prevalence, feature composition and cross-centre generalisability of machine learning diagnostic models for multi-pathogen respiratory infection

**DOI:** 10.3389/fpubh.2026.2015265

**Published:** 2026-10-12

**Authors:** Fengmiao Hu, Xingyu Zhou, Lijun Zhou, Zhirui Li, Shuang Dong, Chongkun Xiao

**Affiliations:** Sichuan Center for Disease Control and Prevention, Chengdu, China

**Keywords:** cross-centre validation, feature composition, machine learning, multi-label classification, multi-pathogen diagnosis, respiratory infection

An incorrect **Funding** statement was provided. The correct **Funding** statement reads:

The author(s) declared that financial support was received for this work and/or its publication. This work was supported by the Sichuan Provincial Center for Disease Control and Prevention Youth Fund Project (Grant No. QN202509).

The original version of this article has been updated.

