# Supplementary material for: Pathogen prevalence, feature composition and cross-centre generalisability of machine learning diagnostic models for multi-pathogen respiratory infection
Source: Front Public Health. 2026 Sep 16;14:1961816. doi: 10.3389/fpubh.2026.1961816 (PMC13623830; doi:10.3389/fpubh.2026.1961816)
Supplement: Supplementary file 1 [file Table_1.DOCX]

**Supplementary File S1. Analysis code and implementation details**

Associated with: Hu F, Zhou X, Zhou L, Li Z, Dong S, Xiao C. Pathogen Prevalence, Feature Composition and Cross-Centre Generalisability of Machine-Learning Diagnostic Models for Multi-Pathogen Respiratory Infection.

## S1.1. Computing environment and pinned library versions

All analyses were performed in Python 3.13 (single-threaded; XGBoost n_jobs = 1; random seed = 42). The exact library versions are pinned below and in the requirements file accompanying the code archive.

```
# requirements.txt  (exact versions used)
numpy==2.5.1
pandas==3.0.5
scikit-learn==1.9.0
scipy==1.18.0
xgboost==3.4.0
lightgbm==4.7.0
catboost==1.2.10
matplotlib==3.11.1
imbalanced-learn==0.14.2   # used only for the SMOTE comparison
```

## S1.2. Data preparation and quality control

The raw surveillance export (24,689 acute respiratory infection records) was cleaned as follows: (i) demographic consistency QC removing 1,133 records with logical contradictions between age and occupation; (ii) exclusion of 2,161 records with any untested pathogen, retaining 21,395 complete-case records. 'Untested' was treated as missing, never as negative.

```
import pandas as pd
df = pd.read_excel('raw_surveillance.xlsx')          # 24,689 rows
# (i) demographic consistency QC (example logic)
contra = (df['age'].notna() & df['occupation'].notna() &
          ((df['age'] < 18) & (df['occupation'].isin(['retired','elderly']))))
df = df[~contra]                                      # -1,133
# (ii) drop records with any untested pathogen
pathogen_cols = [...]                                # 21 columns: positive/negative/untested
df = df[~df[pathogen_cols].eq('untested').any(axis=1)]  # -2,161 -> 21,395
y = (df[pathogen_cols].replace({'positive':1,'negative':0,'untested':0})).astype(int)
```

## S1.3. Feature engineering

Fifteen features available at the time of consultation were used: age, log-age, sex, severity, month, quarter, weekday, day of year, sin(month), cos(month), winter indicator, illness duration, occupation, specimen type, specimen source. Missing values: age -> training median, illness duration -> 0, categorical -> separate 'missing' category, severity -> absent. Calendar month encoded as periodic sin/cos.

```
import numpy as np
df['log_age']   = np.log1p(df['age'].fillna(df['age'].median()))
df['sin_month'] = np.sin(2*np.pi*df['month']/12)
df['cos_month'] = np.cos(2*np.pi*df['month']/12)
df['winter']    = ((df['month'].isin([12,1,2])).astype(int))
for c in ['sex','occupation','specimen_type','specimen_source','severity']:
    df[c+'_o'] = df[c].astype('category').cat.codes
X = df[['age','log_age','sex_o','severity_o','month','quarter','weekday',
        'day_of_year','sin_month','cos_month','winter','illness_duration',
        'occupation_o','specimen_type_o','specimen_source_o']]
```

## S1.4. Model training (per-pathogen one-vs-rest)

For each of the 21 pathogens an independent binary classifier was trained (one-vs-rest). XGBoost was the pre-specified primary model; logistic regression, random forest and a back-propagation neural network were compared under an identical split and threshold-selection protocol.

```
from sklearn.model_selection import train_test_split
from xgboost import XGBClassifier
X_tr, X_te, y_tr, y_te = train_test_split(X, y, test_size=0.2, random_state=42)
def fit_pathogen(col):
    model = XGBClassifier(n_estimators=250, max_depth=6, learning_rate=0.1,
                          subsample=0.8, colsample_bytree=0.8,
                          n_jobs=1, random_state=42)
    model.fit(X_tr, y_tr[col])
    return model
models = {c: fit_pathogen(c) for c in y.columns}
```

## S1.5. Threshold selection and evaluation metrics

The default 0.5 threshold drives minority-class recall to zero, so a per-pathogen threshold maximising F1 was searched on out-of-fold probabilities from 5-fold CV within the training set (range 0.05-0.95, step 0.025) and applied to the test set. Primary metric: Macro-F1 (mean F1 over 21 pathogens). Secondary: group Macro-F1 (prevalence >1% vs <=1%), Macro-AUC, per-pathogen precision/recall/AUC.

```
from sklearn.model_selection import cross_val_predict
from sklearn.metrics import f1_score
def best_threshold(col):
    oof = cross_val_predict(models[col], X_tr, y_tr[col], cv=5, method='predict_proba')[:,1]
    best, bf = 0.5, -1
    for t in np.arange(0.05, 0.95, 0.025):
        f = f1_score(y_tr[col], (oof >= t).astype(int))
        if f > bf: bf, best = f, t
    return round(best, 3)
```

## S1.6. Methodological evaluations

(i) Label-definition sensitivity: Definition A (untested coded negative, all 24,689) vs Definition B (complete case). (ii) Cohort-fixed contamination simulation: within the 21,395 cohort, 10%/20% of samples had 1-2/1-3 originally positive labels recoded as negative. (iii) Leave-one-hospital-out CV: each of 7 hospitals held out as test set. (iv) Prevalence-performance association: Pearson r between log-prevalence and per-pathogen F1. (v) Feature-group ablation: full (15) vs temporal-only (7) vs non-temporal-only (8). (vi) Calibration: Brier score, intercept and slope via logistic regression of outcome on logit(prediction).

```
# (iii) leave-one-hospital-out cross-validation
macro_f1_centres = []
for hosp in df['hospital'].unique():
    te = (df['hospital'] == hosp)
    m = XGBClassifier(n_estimators=250, max_depth=6, learning_rate=0.1,
                      subsample=0.8, colsample_bytree=0.8, n_jobs=1, random_state=42)
    m.fit(X[~te], y[~te])
    preds = m.predict_proba(X[te])[:,1]
    macro_f1_centres.append(f1_score(y[te], (preds>=thr).astype(int), average='macro'))
```

## S1.7. Custom focal-loss objective (XGBoost)

Used for the focal-loss comparison (alpha = 1.0, gamma = 2.0).

```
def focal_loss(alpha=1.0, gamma=2.0):
    def obj(predt, dtrain):
        y = dtrain.get_label()
        p = 1.0 / (1.0 + np.exp(-predt))
        g = alpha * (p - y) * ((1-p)*y + p*(1-y))**gamma
        h = (alpha * (p - y) * gamma * ((1-p)*y + p*(1-y))**(gamma-1) * (p*(1-p))
            + alpha * ((1-p)*y + p*(1-y))**gamma * p * (1-p))
        return g, h
    return obj
# model_focal = XGBClassifier(objective=focal_loss(1.0,2.0), n_estimators=250,
#                             max_depth=6, learning_rate=0.1, n_jobs=1, random_state=42)
```

## S1.8. Permutation importance

Mean Macro-F1 drop when a feature is permuted, averaged over the 21 classifiers and 1,000 random seeds (sklearn inspection.permutation_importance).

```
from sklearn.inspection import permutation_importance
res = permutation_importance(models[col], X_te, y_te[col],
                             scoring='f1', n_repeats=1000, random_state=42)
perm_imp[col] = res.importances_mean
```

## S1.9. Figure generation

All six figures were generated in Matplotlib 3.11.1 with a journal-style template (no in-panel titles; axes and legends only). Fig. 1 workflow; Fig. 2 prevalence-F1 regression; Fig. 3 missing-label strategies; Fig. 4 LOO forest plot; Fig. 5 gain vs permutation importance; Fig. 6 feature-group ablation.
